# P-573. Analysis of Cabotegravir-Rilpivirine Use in HIV Patients at MedStar Washington Hospital Center

**DOI:** 10.1093/ofid/ofae631.771

**Published:** 2025-01-29

**Authors:** Panagiotis Koutsoupias, Glenn Wortmann, Kimberly French, Treatment Navigator

**Affiliations:** MedStar Washington Hospital Center, Washington, District of Columbia; MedStar Washington Hospital Center, Washington, District of Columbia; MedStar Washington Hospital Center, Washington, District of Columbia

## Abstract

**Background:**

Intramuscular cabotegravir-rilpivirine (CAB-RPV) is a long-acting antiretroviral for the treatment of HIV which can be administered every 1-2 months. Registration trials demonstrated the non-inferiority of CAB-RPV versus oral antiretroviral therapy, but data on the ‘real world’ performance of CAB-RPV is sparse. In addition, these trials required HIV virologic suppression (viral load (VL) < 50 copies/mL) with oral medications before transitioning to CAB-RPV. Small studies have reported success when CAB-RPV was started in patients with detectable viremia, and our study sought to build on those findings.

**Methods:**

Descriptive, retrospective study based on patients who began treatment with CAB-RPV between May 2022-Nov 2023. Virologic undetectability (VL < 50 copies/mL), suppression (VL 51-200 copies/mL) and virologic failure (VL > 201 copies/mL) at last measurement are described among patients with a VL < 50 copies/mL, 51-200 copies/mL or >201 copies/mL at baseline.

**Results:**

42 patients are included in the analysis with a median age of 42 (range 27-80). The cohort was 93% African American, 60% male and 81% received Medicare/Medicaid or both as their primary insurer. Median CD4 count was 861 (range 15-1827) and median viral load was 40 copies/mL (range < 20-294,000). Among 38 patients with a baseline VL of < 50, 34 maintained viral undetectability, 1 become virally suppressed (VL=60) and 3 did not have results available due to recently starting CAB-RPV. One patient with baseline viral suppression (VL=90) became virally undetectable. Two patients with baseline virologic failure (VL 1,210 and 7,200) became virologically undetectable. One patient with baseline virological failure (VL=294,000) developed resistance shortly after starting therapy.

**Conclusion:**

Similar to data in registration trials, CAB-RPV maintained viral suppression in 100% of patients who were at least virally suppressed at baseline. Two patients with virological failure at baseline achieved virological suppression, adding to data that CAB-RPV may be beneficial in patients who are unable to adhere to oral antivirals. One patient with baseline virological failure and high viral load quickly developed resistance after starting therapy, supporting data that CAB-RPV needs to be used carefully in certain populations.

**Disclosures:**

**All Authors**: No reported disclosures

